# Hospital Meetings

**Published:** 1903-04-25

**Authors:** 


### HOSPITAL MEETINGS.

### NORTH-WEST LONDON HOSPITAL.

The annual general Court of Governors was held on April 20th, Mr. George Herring, treasurer, being in the chair in the unavoidable absence of Lord Rathmore.

The Chairman called particular attention to the numbers of in- and out-patients treated at the hospital, which were respectively 601 and 17,564, the total number of attendances being 43,674. This large number of cases, said Mr. Herring, was, compared with the size and resources of the hospital, not equalled by any other hospital. As regards the economy of the administration, the secretary of the Hospital Sunday Fund had stated, when paying a visit, that theirs was a model hospital. They had received the large sum of £1,000 from King Edward's Fund, which was double the amount of the last year's grant, and the committee of that Fund had put it on record that the hospital well deserved support.

The report for 1902 having been adopted, a deputation, consisting of two members of the United Friendly Societies, which had made a donation of £31, preferred a complaint as to the treatment of patients furnished with letters from their society, stating that these letters were treated as of no value, and the patients were asked for money. It was said that similar complaints had been made of this hospital on several previous occasions.

The Chairman in reply said that if payment had been asked it was entirely contrary to the rules of the hospital The name and address of one complainant having been ascertained, the Chairman promised that the fullest investigation of the matter should take place so soon as they could learn the date of the occurrence and the identity of the hospital official who had demanded payment.

The report for 1902 showed a slight decrease again in the amount of annual subscriptions. There was an excess of expenditure over income of £1,219. A scheme had been entered upon for obtaining one million pennies in order "to pay Christmas bills," and one-third of that amount had already been raised.

